# A multi‐centre qualitative study of experiences of managing diabetes mellitus among adults while hospitalised

**DOI:** 10.1111/dme.70256

**Published:** 2026-02-18

**Authors:** Sarah E. Mansbridge, Olga Kozlowska, Alistair Lumb, Rustam Rea, Garry D. Tan, Helen Walthall

**Affiliations:** ^1^ Oxford Institute of Applied Health Research Oxford Brookes University Oxford UK; ^2^ Oxford Centre for Diabetes, Endocrinology and Metabolism Oxford University Hospitals NHS Foundation Trust Oxford UK; ^3^ NIHR Oxford Biomedical Research Centre Oxford UK; ^4^ Oxford University Hospitals NHS Foundation Trust Oxford UK

**Keywords:** diabetes, experience, inpatient care, patient‐centred care, patient safety

## Abstract

**Aims:**

One in six hospital beds across England is occupied by someone with diabetes. While guidance on inpatient diabetes care is available, national audit data demonstrate that people still experience significant and avoidable diabetes‐related harms. This study is unique in exploring how people with diabetes admitted to hospital for any medical reason experienced diabetes care from admission to discharge. It is part of a bigger project aiming to develop and test a Patient Reported Experience Measure for inpatients with diabetes.

**Methods:**

A qualitative approach was used to explore experiences of inpatient diabetes care. Twenty‐seven participants with type 1 or type 2 diabetes, hospitalised for any reason, were recruited using purposive sampling across four acute NHS Trusts in the South of England. Data collected in semi‐structured interviews were analysed with reflexive thematic analysis.

**Results:**

In diabetes care, the emphasis is on supporting people with self management of their diabetes. The inpatient care setting compromises this by limiting self management behaviours. These restrictions may apply to those who want to and can be actively involved in their diabetes care and may contribute to less effective diabetes management and poorer outcomes. For some participants in this study, diabetes self management was discouraged in three ways. First, their knowledge of their diabetes and willingness to self‐manage were not taken into account on admission or in planning their inpatient diabetes care. Second, their involvement in decisions about their ongoing diabetes care was limited. Third, their needs related to diabetes management were not met because of the lack of flexibility in hospital practices and schedules.

**Conclusions:**

Inpatient care is not always conducive to diabetes self management. Understanding the patient experience in the inpatient setting related to self management is important in reducing harm to patients while they are in hospital. Our findings emphasise the importance of involving people with diabetes in planning and managing their care while hospitalised. Further work needs to be done to ensure that the knowledge, involvement and flexibility of care of people with diabetes are incorporated into an inpatient setting.

**What's new?**

**What is already known on this topic?**

- Significant and avoidable diabetes‐related harms continue in inpatient care.

**What this study adds?**

- This study uniquely explores how people with diabetes admitted to hospital for any medical reason experience diabetes care from admission to discharge.
- Understanding the patient experience in the inpatient setting related to self management is important in reducing harm to patients.
- Involvement of people with diabetes in planning and managing their care while hospitalised is recommended.

## 1 INTRODUCTION

### 1.1 Inpatient diabetes care

The number of people living with diabetes in the United Kingdom has doubled in the last 20 years, with significantly more being at an increased risk of developing type 2 diabetes in the future.^1^ Over one in six people admitted to hospital have a diagnosis of diabetes (18%), with patients falling into two main groups: 8% admitted for reasons directly related to their diabetes (e.g., diabetic ketoacidosis, hypoglycaemia, hyperglycaemia) and 92% admitted for reasons unrelated to their diabetes (e.g., respiratory infections, cardiovascular events, orthopaedic surgery).^2^ Evidence suggests that people with diabetes are at risk of harm during an inpatient admission. This is highlighted by the England and Wales National Diabetes Inpatient Safety Audit (NDISA)—the successor to the National Diabetes Inpatient Audit (NaDIA)—which collects data on four avoidable complications that can occur in inpatients with diabetes. In 2017, almost 10,000 inpatients suffered a severe hypoglycaemic episode requiring rescue treatment and 2200 people developed diabetic ketoacidosis (Ref. [2] while more recent data is available, it is skewed by the Covid‐19 pandemic).

Several initiatives have been promoted to improve inpatient diabetes care and prevent harms, including multidisciplinary diabetes inpatient teams in hospitals, diabetes‐specific training for healthcare professionals across the hospital, and involvement of people with diabetes in their own care (Refs. [1, 3] reporting on initiatives introduced prior to and after data collection in this study). Particular emphasis has been placed on supporting those hospitalised to self manage their diabetes where appropriate, agreeing a diabetes care plan on admission and providing access to appropriate meals at appropriate times.^1^

### 1.2 Principles of inpatient diabetes care

The management of long‐term conditions such as diabetes requires collaborative management strategies between healthcare providers and people living with the condition. These strategies form the basis of effective self management and ensure all aspects of managing a chronic illness are addressed.^4^ The importance of self management for people with diabetes and other long‐term conditions is well documented^5^; the education programmes for people living with diabetes (e.g., DAFNE, DESMOND and X‐PERT) are structured to empower individuals to take ownership of and actively manage their diabetes (what to do if glucose readings are outside the target range, how to adjust diet, exercise, lifestyle, medication and insulin). People with diabetes have been shown to become experts in the management of their diabetes with biomedical, experiential and practical knowledge exceeding that of many healthcare professionals.^6^

There is extensive evidence to support how self management can be effective in the management of chronic illness including diabetes,^7^, ^8^, ^9^ yet this is predominantly in the primary care setting.^10^ Regarding inpatient hospital care, there is guidance available. The Joint British Diabetes Societies for Inpatient Care Group promotes people with diabetes continuing ‘to self‐manage during a hospital admission unless there is a specific reason why they cannot’^11^ and Diabetes UK outlines support for self management in the hospital setting,^1^, ^11^ yet how and when this has been implemented is less well documented, with some evidence emerging from across the country.^12^ The NaDIA patient experience survey, reporting on inpatients' views of their hospital stay, rated some aspects of diabetes care as poor, suggesting the diabetes inpatient guidance is not fully followed. For example, patients have described dissatisfaction with meal choices, a lack of ability to self‐administer insulin, no control over blood glucose management and no involvement in their care planning.^2^

Broader than diabetes care, the analysis of inpatient hospital care in England revealed that the most significant problem in relation to self management was the failure to provide active support for patient engagement, and it was noted that care can still be delivered in a paternalistic manner.^13^, ^14^ Despite overwhelming evidence suggesting that a patient's experience of hospital care and being involved in their care translates into well‐being and recovery^13^, ^15^ and can help to avoid a medical emergency,^1^ supporting patients to self‐manage appears not to be prioritised.

The findings outlined from the available evidence including national audits, and the need to support self management in diabetes for those who have been admitted to hospital, prompted our qualitative study to explore in depth how people experience diabetes care during the episode of care from admission to discharge. Our study set out to answer the following research question: ‘*How was diabetes self management supported in an acute inpatient care setting?*’ Understanding the patient experience of diabetes self management in the inpatient setting offers a potential route to improving patient experience of care and reducing diabetes‐related harm in the inpatient setting.

## 2 METHODS

To explore experiences of managing diabetes while hospitalised, a qualitative approach was used. The *Consolidated criteria for Reporting Qualitative research* * checklist^16^ was applied to report on this research.

### 2.1 Recruitment

Participants were recruited from inpatients in four acute NHS Trusts in the South of England with a diagnosis of diabetes mellitus. These were acute teaching trusts providing dedicated inpatient diabetes care and included: a large trust with approximately 1300 beds spread across 4 hospital sites; a large trust with its main acute hospital having approximately 813 beds; a medium‐sized trust consisting of one general hospital with approximately 550 beds; and a medium‐sized trust providing integrated hospital and community services with acute services across two hospitals with approximately 619 beds.

Purposive sampling was used to capture the experiences of women and men across different age groups, using a range of oral and injectable treatments and being admitted via planned or unplanned routes. People with type 1 and type 2 diabetes who were admitted for any episode of care and for any cause were included. Inclusion criteria were as follows: patients (1) diagnosed with diabetes mellitus before the hospital episode, (2) with a hospital inpatient episode (involving an overnight stay) for any reason and (3) aged 18 years or older. Patients with (1) other types of diabetes, (2) cognitive impairment, (3) receiving end‐of‐life care, (4) residing outside the study region or (5) unable to speak or read English were excluded from the study. Those with other types of diabetes were excluded because the planned sample size meant that some types of diabetes would not be represented, and where a rare type of diabetes was represented, the experience would be limited to a very small number of people, potentially skewing the results.

The diabetes clinical team identified patients meeting the inclusion criteria and sought their agreement to be contacted by a research nurse. Potential participants were approached by the research nurse at the time of discharge to discuss the study. A research pack was also given to them that included a letter of invitation, a Participant Information Sheet, a reply slip and a prepaid envelope. Potential participants were advised to familiarise themselves with the research pack and, if they were considering participating in the study, to return the reply slip in the prepaid envelope. On receipt, potential participants were contacted by a member of the research team (S.M.) to arrange a convenient time and place to conduct the interview.

The sample size was estimated at between 20 and 30 participants; the sample size considerations included the breadth and focus of the study (experiences of inpatient diabetes care) and the diversity of the population (type of diabetes, type of admission, age and sex). The expectation for the semi‐structured interviews was to generate rich data. The richness and complexity of the data were the guiding principles when deciding to stop recruitment.

### 2.2 Data collection

The interview schedule was designed to capture all aspects of participants' diabetes care from hospital admission to discharge (Appendix 1). The interviews were semi‐structured to ensure the same questions were asked to all participants, whilst also allowing them to include experiences that were of personal relevance to them. The interview schedule was informed by the literature on managing and self managing diabetes and views of the project's advisory committee. The interview schedule was piloted on the first two participants, with the interviews included in the dataset, which resulted in one question being added (How were you admitted?).

All interviews were recorded using a digital voice recorder and were later transcribed verbatim. O.K. checked the accuracy of each transcript against the original audio recording and de‐identified the transcripts.

### 2.3 Researchers' characteristics and reflexivity

The research team members working with data included S.E.M., H.W. and O.K. All are doctoral‐level trained and experienced qualitative researchers. The researchers were independent of the data collection sites and there was no conflict of interest. S.M. and O.K. have backgrounds in psychology. H.W., A.L., R.R. and G.D.T. are clinical academics. H.W. has clinical expertise in cardiovascular and adult nursing and A.L., R.R. and G.D.T. have clinical expertise in acute and diabetes care. The interviews were conducted by S.E.M.; S.E.M., O.K. and H.W. analysed the collected data and feedback was then sought from the full team.

### 2.4 Reflexive thematic analysis

To explore experiences of diabetes care during an inpatient stay, we employed reflexive thematic analysis.^17^ We were interested in uncovering the role people with diabetes expected, and were expected, to play while hospitalised. Critically, we wanted to find out how they negotiated the management of their diabetes care and their involvement in decisions made about this. Thus, thematic analysis was selected for use in the current research, as it allowed us to identify patterns of meaning in the collected data, which provided important insights into the above issues. The analytical process was both inductive and deductive; the Picker principles of patient‐centred care^18^ provided a broad analytical framework but without restricting the codes.

The interviews were uploaded into NVivo (QSR International) for thematic coding by S.M., H.W. and O.K. We followed the six‐stage process of thematic analysis^19^; initially we familiarised ourselves with the data by listening to, reading and re‐reading the interviews. Next, we systematically coded data for content (what the participants recalled about their diabetes care at different stages of the hospital stay). We then generated the initial themes from coded and collated data related to the participants' perceptions of their role in their own diabetes care; the themes were subsequently developed and reviewed in an iterative process to be eventually defined and named. We regularly discussed and agreed on code labels and themes.

### 2.5 Patient and public involvement

A member of the Patient and Public Involvement Group at the Oxford Centre for Diabetes, Endocrinology and Metabolism was part of the project's advisory committee and helped to shape the design of the study, its management and analysis of results, providing feedback throughout the process of development of the themes.

### 2.6 Ethical approval

The project was approved by the Research Ethics Committees at the Faculty of Health and Life Sciences, Oxford Brookes University (no.: 2018/13) and the NHS Health Research Authority (no.: 19/LO/0644). This research is part of a bigger project aiming to develop and test the Patient Reported Experience Measure for inpatients with diabetes; the interview stage was the first stage in data collection to inform content of the new tool. Informed consent was obtained at the time of interview, after giving participants the opportunity to ask any further questions.

## 3 RESULTS

### 3.1 Participant characteristics

Twenty seven participants were recruited and consented to be interviewed. Recruitment stopped when the sample included representatives of all clinical and demographic characteristics (the type of diabetes and admission, sex and age); by this stage, no new perspectives were being elicited from the interviews. Table 1 shows how our sample compared with the population of inpatients with diabetes in England and Wales 2017.^2^

**TABLE 1:** Participants' characteristics.

Category	Sample characteristics	Population of inpatients with diabetes characteristics
Type of diabetes (at the time of admission)	Type 1: 7 (25%)	Type 1: 10%
	Type 2 insulin: 20 (29.5%)	Type 2 on insulin: 30%
	Type 2 non‐insulin: 10 (37%)	Type 2 non‐insulin: 40%
	Type 2 diet only: 2 (7.5%)	Type 2 diet only: 20%
Type of admission	11 elective (41%)	Elective: 20%
	16 non‐elective (59%)	Non‐elective: 80%
Sex	15 M (55.5%)	Not available
	12 F (44.5%)	
Age (years)	Between 29 and 88, average of 60.5	Not available

### 3.2 Data collection

The interviews were conducted between August 2019 and February 2020 within 2 weeks of discharge. Participants were primarily interviewed at their home address; one participant was interviewed on the phone as this was their preference. Interviews lasted between 30 and 90 min.

### 3.3 Findings

Participants differed in their expectations about their role in managing diabetes while in hospital, with some expecting to be involved and others not. Among those who did not expect to be involved in managing their diabetes were those who were hoping inpatient care would provide them with a respite from the everyday burden of self‐managing diabetes, those who were not expecting any diabetes care would be provided to them as their admission was not diabetes‐related and those who were anticipating and accepting of hospital care to be clinician‐led. Experiences of those who did not expect to be involved in managing their diabetes were not the focus of the analysis; however, they are important to mention to provide a fuller picture of how inpatient diabetes care is being experienced and to recognise diverse cases within the topic.

**FIGURE 1:**
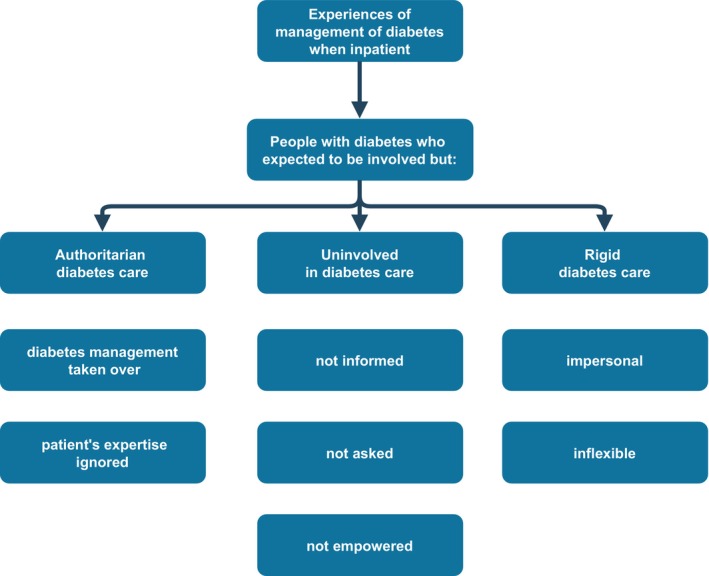
Themes developed from the interviews.

Those who expected that they would be involved in managing their diabetes reported some experiences of being sufficiently involved in aspects of their diabetes management; however, negative experiences of not being involved dominated in this group. These were related to three types of situations: when clinicians (nurses and doctors) took control of a person's diabetes care (theme one: *authoritarian diabetes care*), did not communicate with them about their diabetes care (theme two: *uninvolved in diabetes care*) or allowed for the rigid hospital practices to override their needs (theme three: *rigid diabetes care*). The focus of the analysis in this paper is on those who expected to but were not involved in their diabetes care; this is because the clash between what people with diabetes expected and what was expected of them in managing their diabetes has contributed to a poor experience of inpatient diabetes care. Figure 1 represents the final themes and subthemes.

### 3.4 Theme 1: Authoritarian diabetes care

#### 3.4.1 Subtheme: Taking over management

On admission to hospital, in some cases clinicians assumed control over the management of diabetes (e.g., monitoring their blood glucose levels, administering their medication). Some saw this as respite from their diabetes self management; they had confidence in the healthcare team and were happy to relinquish control:

Delighted. Well they knew what they were doing and, you know, if you go to people who know what they're doing, you trust them. You know, let them get on and do their job. (Participant 2; male, 72, T2D, elective admission—kidney stones)

Others, however, wanted to continue to self‐manage. They perceived the loss of control as ‘belittling’ or ‘degrading’, as they had often been managing their own diabetes for a considerable time. Indeed, they felt they had greater understanding of what was best for them (and their own bodies) than the healthcare team; further, they did not always have confidence in the clinicians looking after them, particularly the general nursing staff:

[…] I was [treated] like a little child that didn't know what they were doing and, you know, couldn't be trusted to know what was best for me, which is degrading really. (Participant 3; female, 63, T2D, elective admission—knee surgery followed by non‐elective admission due to the post‐surgery infection)

So this nurse said to me, well if you're taking any insulin, I would like to see you drawing it up and administering. I have drawn up and administered more doses over the last fifty years than you have done in your one or two years training. But that was about not trusting the patient to have the skills, the ability and the knowledge to be able to do something as simple as dialling up a dose. (Participant 4; male, 62, T1D, non‐elective admission—urinary tract infection)

#### 3.4.2 Subtheme: Ignoring patient's expertise

The expertise of a person with diabetes was often ignored by clinicians when offered and had the effect of them not feeling listened to. For example, if they tried explaining that their blood glucose levels were high because their bloods had been taken at an incorrect time for them (e.g., after eating rather than before):

The information that I gave would have been helpful but it wasn't listened to. No notice was taken […] but I'm the expert about my diabetes not her [the diabetes nurse specialist]. (Participant 3; female, 63, T2D, elective admission—knee surgery followed by non‐elective admission due to the post‐surgery infection)

Furthermore, clinicians did not ask for a person's input when needed. For example, why they could have suddenly experienced high or low blood glucose levels. Participant 7 shared his experience of being cared for by a team who did not consult with him regarding his diabetes treatment which, according to him, led to a hypoglycaemic episode:

And then I got moved… still not knowing why I'd gone into a hypo… But once I got there and the lady came, […] she said, do you know what's been going on? And I said […] the only thing I can think of is they've been giving me the pills but not checking that I'm eating and I'm drinking the amount what I should be to use them tablets. They went, oh right, OK. Well yes, that's a fair comment. (Participant 7; male, 60, T2D, elective admission—back surgery)

People with diabetes were sometimes also dismissed if they challenged clinicians' decisions about the management of their diabetes care and felt they had to fight to be heard:

But I did have another issue with the consultant in the actual A&E, trying to send me home after we had made […] this plan of what was going to happen […] with the consultant, with my obstetrics doctor and the diabetes nurse. And then when they had gone, so when they had gone home, so he couldn't call them anymore, about half six at night, another A&E doctor came in and said, well I don't think you're sick enough, you don't look sick to me, you can go home. So I was like, well one minute, how am I meant to look sick? I'm diabetic, you don't really look sick when you're diabetic. I mean unless you're in like serious DKA or having a hypo, you shouldn't be looking that sick. So he tried to send me home, to which I had to tell him, no, I'm not going home. And he sort of stormed out and had a tantrum about it. (Participant 16; female, 29, T1D, non‐elective admission—hyperglycaemia)

### 3.5 Theme 2: Uninvolved in diabetes care

A further characteristic of participants' experience was that they felt acted upon, rather than involved in the decisions made about their ongoing diabetes care.

#### 3.5.1 Subtheme: Not informed

Clinicians did not always consult a person with diabetes when changes were made to their medications (e.g., new ones started or current ones stopped), which left them feeling ‘upset’ and ‘annoyed:’

Well as soon as you get there they ask what tablets you're on but they never gave me any Metformin tablets. So they were just managing it with the insulin […] but I thought that was a bit strange, because I always take two in the morning and two at teatime, and they didn't give me any of those. […] And I asked, I asked, oh I haven't had these tablets. And she wrote it down and went off and didn't come back and say anything. (Participant 17; female, 55, T2D, non‐elective admission—diabetic ketoacidosis)

Oh no, I wasn't told. I had to ask, where's my Metformin tablet? And then I was told, well you've not been written up for it, so doctors, obviously, don't feel that you need it. […] I was a little bit annoyed at first, that they hadn't told me, but then when I realised that everything was going on the same without them, I thought, oh well maybe I didn't really need them. (Participant 24; female, 63, T2D, non‐elective admission—severe fluid retention)

#### 3.5.2 Subtheme: Not asked

Participants also cited that they were not included in decisions made about the management of their diabetes during their hospital admission. Indeed, their preferences were not taken into consideration by the healthcare team. For example, they were not asked if they wanted to continue to self‐manage, or if they would like assistance from clinicians, family or friends:

I don't think I was ever asked. I was told that if I needed support I could have support. In other words, they assumed that I would take control and if I needed help I'd get help. That was the feeling I got. (Participant 26; female, 73, T2D, elective admission—cancer treatment complications)

#### 3.5.3 Subtheme: Not empowered

The healthcare team rarely, if at all, discussed patients' diabetes care with them. Some participants did not expect or want this. For example, if they had been admitted for a non‐diabetes‐related issue or were not experiencing any difficulties controlling their diabetes:

Well, I guess, I thought that, to be honest, with regards to my diabetes, I knew that I had my regular check‐up a couple of weeks or a month, whenever, before I had this accident, for want of a better description. So, you know, I'm under the impression that that's being managed by my GP surgery. And so I wasn't really looking for the nurses in the hospital to intervene. (Participant 18; male, 57, T2D, non‐elective—kidney stones)

No. On the day that I left, I think they had a few emergencies, because they moved me from where I was into a separate ward, and then I was discharged from there. So no further discussions at all on diabetes. […] I don't think it would have made any difference because I don't think at the time that that was forefront in my mind, to be honest. […] It was more a cardiac related admission and the diabetes was really, not irrelevant, but low priority, shall we say, for me. (Participant 23; female, 71, T2D, non‐elective—arterial thrombosis)

Other participants, however, had questions or concerns about their ongoing diabetes care and were expecting, or would have liked, to have spoken with a member of the healthcare team before going home:

No. The doctor said, you can go home, and then I was just waiting and waiting for a nurse to become available to take my cannulas out, that was it. […] And I was ready to go but I wasn't ready because I didn't know what I was doing. So I could have, I should have had somebody there I feel, to tell me how to manage [diabetes] at home. (Participant 17; female, 55, T2D, non‐elective admission—diabetic ketoacidosis)

### 3.6 Theme 3: Rigid diabetes care

The final theme examines how the rigid (the same for all patients with diabetes) procedures implemented in the hospital setting affected the experience of diabetes care.

#### 3.6.1 Subtheme: Impersonal

Participants referred to the rigid procedures implemented in the inpatient setting as having created an impersonal environment, which was not always sensitive to their emotional needs. For example, some participants reported that they had not been allowed to keep their insulin with them. This had evoked strong feelings of ‘vulnerability’ as they were dependent on it to keep them alive:

I was asked to disclose what medicines I had with me, which I did, I handed over a lot of them […] but I wanted to retain my insulin. Diabetics have a very close relationship with their insulin, it's what keeps them alive […] I like to have that with me at all times because it may well be that I will do a blood test that will show that my blood sugar levels are too high, and so I might want to take a corrective dose. (Participant 4; male, 62, T1D, non‐elective admission—urinary tract infection)

Participants also described the routine taking of bloods every few hours as a ‘tick box’ exercise, with clinicians seemingly going through the motions. When asked about her routine of blood glucose level testing before being admitted, Participant 21 said:

I do occasionally, but after thirty eight years, you know how you feel. So not four times a day, every day [as in the hospital]. My fingers were actually, never ever have I had bruised fingers from blood testing, but my fingers were actually black and blue all along the tips. It didn't seem to make a lot of difference to the results. (Participant 21; female, 65, T2D, non‐elective admission—hypoglycaemia)

This also led participants to perceive that procedures were being carried out at inappropriate times. For instance, they often commented that they had been disturbed or woken during the night for bloods:

They started off every hour, through the night every hour. And then it got to Friday night, they started every hour, woke me up at about midnight and did it, and then that woke me up, so I was awake, and they didn't come back, they didn't come and do it again. So I thought, 2 O'clock, are they going to come? No. And then I was still awake at 4 O'clock and they hadn't been. And I thought, they come when you're asleep and wake you up but when you're awake they leave you alone. (Participant 17; female, 55, T2D, non‐elective admission—diabetic ketoacidosis)

The hospital setting also created a degree of inflexibility, which further challenged people's management of their diabetes. This is discussed in more detail next.

#### 3.6.2 Subtheme: Inflexible

The hospital environment dictated that patients had set mealtimes, rather than being able to eat when they wanted or needed to (i.e., to avoid their blood glucose levels falling too low). Some people with diabetes experienced difficulties getting hold of food outside these times; they resorted to such measures as thinking ahead and saving food, such as a biscuit from their lunch, which they could then eat later:

But from my diabetes point, it was well controlled, apart from when I literally went to bed and my sugar levels dropped. Because, it sounds weird but when I'm at home, it was more controlled because I could just snack. And when I'm at work I just snack, literally, all through the day. But once you're asleep in the hospital, there's no way of getting anything […] well there was, you had to try and buzz for a nurse, but they were so, literally, just short staffed, to get something to eat. So that's why I, literally, stocked up on all my snacks during the day time. (Participant 8; male, 55, T2D, non‐elective admission—an infected foot ulcer)

Furthermore, participants also remarked that hospitals did not cater particularly well for people with diabetes. Indeed, menus contained very few suitable options for them to choose from:

That is one thing that I did think was strange. Now, when I go into hospital in [country], they give me a choice of a diabetic menu. Here, no choice, you know, you can't have ice cream, you can't have jelly, you know, there wasn't brown bread, there wasn't the things that a diabetic should have on the menu […]. And when ice cream was there, yes, I took it, because I don't have ice cream here and I do love ice cream. And I thought, you know, while I'm here, ice cream's not going to hurt me. But nobody was saying to me, you know, here's the diabetic menu or, you know, it didn't show on the menu, this is a diabetic one, this is a vegetarian one, you know like when you go to a restaurant […]. The food was lovely, don't get me wrong, it was nice, but I did feel some guidance could have been better managed there, definitely. (Participant 22; female, 56, T1D, non‐elective—Legionnaires' disease and sepsis)

It is worth noting that people with diabetes were sometimes unsure what food choices were suitable for them, as this was not always indicated on hospital menus (i.e., with a ‘D’ for diabetes).

These findings suggest that the hospital setting made it more difficult for people to manage their diabetes with diet.

## 4 DISCUSSION

### 4.1 Inadequate support for diabetes self management in inpatient hospital care

The present study aimed to examine patients' experiences of managing their diabetes while hospitalised. Although there is guidance available on supporting people with self management of their diabetes,^1^ the data in our study uniquely focused on experiences of people with diabetes indicate that inpatient diabetes care is still compromised by limiting self management in those who want to be actively involved. Expert, independent and empowered patients expected to be ‘part of the healthcare team’, as one of the study's participants emphasised. However, what we heard was that self management was discouraged for some in the hospital setting.

Three themes were generated from our data coming from this group of people with diabetes: ‘*authoritarian diabetes care*’, ‘*uninvolved in diabetes care*’ and ‘*rigid diabetes care*’. Our findings suggest clinicians simply took control of diabetes care without first consulting with a person with diabetes about their current diabetes management plans and preferences while in the hospital. Furthermore, clinicians appeared not to recognise people's own expertise in managing their condition; this had an undermining effect on some people. We also found in the second theme, *uninvolved in diabetes care*, that clinicians made decisions about a person's ongoing inpatient diabetes care without involving them. This caused some people distress, as they had no opportunity to raise concerns they may have had or clarify how their self management should proceed once back home. Theme 3, *rigid diabetes care*, indicated that the systematic diabetes care that is typical to a hospital setting created challenges for them in managing their diabetes (e.g., difficulties obtaining food outside set mealtimes). Indeed, people with diabetes had to adjust how they typically managed their diabetes to maintain some control while in the inpatient setting (e.g., taking their insulin after they had eaten rather than beforehand). Furthermore, some of the procedures implemented while hospitalised led to people experiencing emotional distress (e.g., having their insulin taken away from them), which seemingly went unaddressed by the healthcare team. To summarise, people with diabetes felt reduced to passive recipients of care that were simply acted upon, rather than being recognised as an active and integral part of the healthcare team. Some clinicians failed to empower people with diabetes in the self management of their diabetes, and instead actually evoked a state of dependence in them. These themes mirror the dimensions of care valued by patients, namely patient–professional interactions, communications and being treated as an individual,^14^ and from diabetes‐specific literature, providing information, monitoring and responding quickly, and listening.^20^ Our findings resemble those from more than a decade ago when the inpatient hospital care approach was deemed paternalistic.^14^ Studies into patients' experiences of inpatient diabetes care from other countries indicate that England is not alone with these problems.^21^, ^22^

### 4.2 Challenges to diabetes self management in inpatient hospital care

There is evidence that having learnt how to self‐manage successfully, people living with diabetes find it confusing, frustrating and frightening to be denied access to self management resources.^6^ Indeed, the mismatch between the specialist diabetes guidance and this study's participants' experience is clear. Our study's data suggest that the challenge of empowering people to self‐manage their diabetes while an inpatient can be considered at three levels of organisation of care, namely micro, meso and macro level.

On the micro level, some people with diabetes felt silenced at the point of admission when the control over their diabetes shifted from the person with diabetes to clinicians (nurses and doctors). On the meso level, people with diabetes felt subjected to care practices habitual to the team on the ward—‘this is how we do it here’. On the macro level, diabetes care was system driven, with the rigid hospital procedures and policies in place—‘this is how a hospital functions’. People with diabetes were exposed to a network of interlinked agents (individual clinicians and systems) where actions that felt oppressive were magnified by being ingrained on all levels of the inpatient care setting. Even if self management was enabled on one level (e.g., clinicians allowed for self‐managing diabetes with insulin) it might have been disabled on another level (e.g., meal choices due to hospital's catering).

The three themes identified in this study had a thread running through them—an overarching theme of the ‘*traditional clinician‐patient roles*’, which implicitly operated within the hospital setting. The moment of entering the hospital was equivalent to a shift in control from patients to the healthcare team—a paternalistic approach to diabetes care was enacted despite available guidelines. The voices of people with diabetes, powerful outside of the hospital walls, were ignored. While the traditional clinician–patient roles and institutionalisation of care, with patients expected to obey clinicians, have been exposed as neither acceptable nor effective,^23^, ^24^, ^25^, ^26^ our data suggest that the inpatient setting is slower to be penetrated by the ideas of patient empowerment. One of our participants expressed it particularly well, ‘My views did not matter because decisions had been made. A patient came second to the system. (I was told) “We have protocols and we are not going to change it for one person”.’

### 4.3 The impact of the contradictory narratives about self management on care

The double standard in diabetes care, promoting self management among people with diabetes at home only to take the control away from them when hospitalised, sends a conflicting message to people with diabetes and hospital staff. Taking away involvement in one's own diabetes care may result in compromised diabetes care when in a hospital and after discharge.

First, the patient's safety may be put at risk, with the severity of risk influenced by the type of diabetes and treatment for diabetes. While for some the interruption to their usual diabetes care during a short hospital stay may not have a lasting impact on their diabetes‐related health outcomes (although it may carry different types of risks as explained next), too many people experience diabetes‐related life‐threatening complications.^2^, ^27^ Moreover, what is planned as a short inpatient stay may be followed by re‐admission(s) due to diabetes or non‐diabetes‐related complications of the inpatient stay. Self management while an inpatient may help to prevent poor diabetes outcomes when in hospital; for example, self‐managing people with diabetes receive insulin at the appropriate times and doses.^28^ People supported to self‐manage diabetes when in hospital should also be less likely to be readmitted due to post‐discharge diabetes complications.^29^ However, self management should not be seen as an expectation for all, as some people with diabetes do not want to or cannot self‐manage due to the severity of the condition they are hospitalised for (e.g., Ref. [30] on complexities of self management of diabetes by inpatients).

Second, conflicting messages that people with diabetes receive in different care settings may undermine their trust in healthcare professionals and prevent their further engagement with healthcare services.^31^ Developing a fear of being admitted to a hospital again and feeling discouraged from engaging in one's diabetes care when readmitted should be considered.

Third, preventing a person with diabetes from participating in their care and self‐managing their condition may lead to a change in how they perceive the nature of the condition and their control over it.^32^, ^33^, ^34^ Those empowered to self‐manage diabetes attribute their health outcomes to their own actions; prolonged and repeated situations of this control being taken away should be considered as leading to a person starting to doubt themselves and relying on others.

### 4.4 (Clinical) implications

The findings reinforce the value of listening to people with diabetes during a hospital stay to improve their individual experience—ensuring they are informed and in control of their diabetes with medication and diet. This is especially important for those whose stay is prolonged or recurrent.

This emphasises the importance of breaking down traditional clinicia–patient roles through empowering (rather than disempowering) a person with diabetes by:

- Consulting with people with diabetes on their diabetes and recognising their expertise
- Involving people with diabetes in decisions about their ongoing diabetes care—including or viewing them as a valued member of the healthcare team.
- Educating people with diabetes on diabetes^35^; our data showed lost opportunities to help people with diabetes understand their condition.
- Ensuring people with diabetes have a clear self management plan on discharge (e.g., Ref. [36]) and are well trained on any new diabetes equipment etc.

The findings reinforce the importance of exploring patients' experiences (how patients feel about their care). The lack of systematic feedback from patients (e.g., national or local audits) can falsely reassure clinicians that the care they offer is right and best for patients. Collecting information on clinical outcomes is not sufficient as only exploration of patients' experiences enables one to understand if all outcomes are relevant, what outcomes are missing, and why outcomes are poor. The National Diabetes Experience Survey† gives people living with diabetes the opportunity to share their experience of NHS care; however, it has not as yet reported on inpatient care. Measuring the experience of inpatients as part of the annual National Diabetes Audit should be a standard of care. An inpatient‐specific patient‐reported experience measure, for example, one developed by Kozlowska et al.,^37^ can assess experiences of diabetes care during hospital inpatient admissions and be used for auditing, evaluating and improving quality of inpatient diabetes care.

Future research should explore the health outcomes of temporary disempowerment of people with diabetes over time (during an inpatient episode, after the discharge and long‐term impact). More research is needed to identify effective strategies to enable self management of diabetes in the inpatient care setting; this includes how to capture the knowledge, preferences and flexibility in the electronic health record, how to communicate this across healthcare records, how to enable the patient to input into this ‘care plan’ and how to give the flexibility of care that some patients prefer in a hospital setting.

### 4.5 Strengths and limitations

The study recruited from a limited number but diverse sites and patient populations; the sample was representative of the population of inpatients with type 1 and type 2 diabetes and their different demographic and clinical characteristics. We are aware that excluded groups of patients with diabetes were not represented and their experiences need separate consideration (patients with other types of diabetes, cognitive impairment, receiving end‐of‐life care or unable to speak or read English). This study elicited and analysed patients' voices in a systematic and rigorous way. The analyses recognised the complexity of experiences including the alternative narratives with inpatient diabetes care. We cannot claim that the study identified good or bad practices related to self management of diabetes in inpatient care; indeed, we collected patients' perspectives only and cannot comment on participants' judgement of to what extent self management was possible and clinically advisable. However, we feel confident that the themes identified are pertinent to inpatient diabetes care.

## 5 CONCLUSION

To our knowledge, this paper is unique in exploring how people with diabetes experience diabetes care while hospitalised for any medical reason. While there are data available on outcomes of inpatient diabetes care and guidance on how to improve these outcomes, preventable hospital‐induced diabetes‐related harms still occur. This paper offers insight into the reasons why this may occur and emphasises the importance of listening to people living with diabetes in seeking answers and solutions to experiencing inadequate inpatient diabetes care.

## CONFLICT OF INTEREST STATEMENT

None.

## Footnotes

*
https://bmjopen.bmj.com/content/suppl/2015/10/19/bmjopen‐2015‐008847.DC1/bmjopen‐2015‐008847supp.pdf.

†
https://diabetessurvey.co.uk/.

## APPENDIX 1 INTERVIEW SCHEDULE

1. Can you tell me why you were recently admitted to a hospital? How long was your stay? How were you admitted?
2. Although you were not admitted to the hospital to treat diabetes, we are interested in your experience of being a patient with diabetes when in hospital. Thinking about your diabetes, how was the hospital stay for you?
  - Did anybody talk with you about your diabetes when you were admitted? At some point later? What were you asked? What were you told? Were things agreed with you? Was it helpful? In what way was it helpful or unhelpful?
  - Before your hospital stay, did you monitor your sugar levels? Was the way you monitored your sugar levels when in the hospital any different from what you usually do? Was it better or worse?
  - Were you asked if you would prefer to manage your diabetes yourself with support from the clinical team?
  - Before your hospital stay, were you doing anything to manage your sugar levels? Was the way you managed your sugar levels when in hospital any different from what you usually do? Was it better or worse?
    - Do you take insulin? And when in the hospital…?
    - Do you take other medication for diabetes? And when in the hospital…?
    - Do you follow a diet? And when in the hospital…?
    - Do you do anything else? And when in the hospital…?
3. If you get assistance with managing your diabetes at home, were you given the option of involving others during your admission?
4. Did someone talk with you about your diabetes on discharge? What was discussed?
5. What happened to your sugar levels monitoring and management after you came back home?
6. In overall, what impact do you feel the hospital stay had on you in terms of your diabetes?
7. Is there anything else that we did not talk about and you think is worth mentioning? Are there any other aspects of hospital stay that are important to someone who has diabetes?
